# New products

**DOI:** 10.1155/S1463924686000433

**Published:** 1986


Journal of Automatic Chemistry/*Journal of Clinical Laboratory Automation*, Vol. 8, No. 4 (October–December 1986), pp. 216–220

### New products

### Sugar Analysis System

A system for the determination of carbohydrates using patented post-column reaction technology with high pressure liquid chromatography, has been launched by Severn Analytical. The Sugar Analysis System measures both reducing and non-reducing sugars simultaneously and utilizes a new combination of heterogeneous chemistry. The system allows sensitive detection of all the common carbohydrates with the minimum of sample preparation; in most cases all that is required is filtration.

At the heart of the system is the 'Chem-Phase' catalytic reactor, a solid-phase system which converts non-reducing sugars into their corresponding monosaccharides. This process is known as inversion; in liquid medium, the process is not very efficient. On the Chem-Phase reactor, which is held at 85°C, the reaction goes to completion. Sucrose, a non-reducing disaccharide, is converted into glucose and fructose, both reducing sugars. As the Chem-Phase reactor is catalytic, it is not consumed and can be expected to last as long as the analytical column. After catalysis, the reducing species are derivatized by the sugar reagent, 4-amino benzoyl hydrazide. The reagent reacts quantitatively to give a reaction product that absorbs strongly at 405 nm.

Whilst the 'Sugar System' is specific, it consists of a complete liquid chromatograph, which has the capability to perform a number of related assays such as organic acids, anions, pesticides, vitamins and food additives.

A novel part of the system is the provision of a guaranteed methodology, which includes on-site installation, training and all the required reagents.

*For further technical information contact Rob Jones, Severn Analytical, 36 Brunswick Road, Gloucester GL1 1JJ, UK.*

### Compact emission spectrometer for fast, economical metals analysis

The PV 8030 high-performance emission spectrometer from Philips Analytical combines dedicated-instrument economy with much of the flexibility of individually-tailored systems for fast, accurate analysis of irons and steels.

Full 1 m optics deliver higher resolution than other instruments in its class. And rugged construction, plus efficient temperature control and vibration-proof mountings, ensure stable operation in an industrial environment.

Two factory calibration packages cover all the most important elements in low-to-medium alloy steels, or irons and steels. Remaining capacity may be filled with elements to match the majority of users' requirements.

A choice of two programmable monoalternance source units – a 50 Hz or a high repetition 50–500 Hz version – can be fitted. the variable frequency unit allows determinations to be completed in 4 s.

A high-energy preburn can be programmed to deal with inhomogeneous samples, such as cast iron. With the high repetition unit, discharge parameters can be varied during the course of a single measurement. Therefore it is possible to optimize accuracy for major constituents and detection limits for trace elements, without undertaking separate analyses.

Microprocessor electronics monitor all key spectrometer functions and warn of any operating error. Additional software in the microprocessor memory improves measuring efficiency. Measurements are composed of a series of mini-integrations, the digitized values of which are accumulated in the microprocessor. This results in a remarkable dynamic range of over 1 million : 1. Another unique feature is the fact that a pre-selected number of complete discharges is always measured – thus assuring optimum repeatability.

Specially designed solid-state circuitry greatly improves counting speed and reliability – allowing extremely accurate measurement down to the very low light levels necessary for trace analysis.

The built-in microprocessor operates in conjunction with a Philips PC, making it easy to use the spectrometer's advanced analytical capability. The powerful Philips software package uses menus and dialogues to guide the operator through every stage. Complete measuring routines can be stored and then recalled by a single command, so shop-floor personnel can easily handle all daily procedures.

An optional software package is also available for DEC computers supporting the RSX-11M operating system; operator communication is structured to match that of Philips X-ray spectrometry software.

*More information from Philips, Industrial and Electro-acoustic Systems Division, I and E Press Office, HKF, Eindhoven, The Netherlands. Tel.: 040 788620.*

### New sample handling for RA-1000 retains IDEE

A new sample handling option for the Technicon RA-1000 family of instruments retains the facility for positive sample identification. The new 'Combi-tray' unit can be retrofitted to existing systems and gives the ability to use original blood tubes and many currently available micro-sample tubes, in addition to standard sample cups.

With the positive sample identification facility the system may be controlled by Technicon's own Instrument Data Manager, or other data-processing systems, without the need for rigidity associated with worklists, allowing automatic rescheduling of dilutions and re-runs, and easy 'stat' insertions. With the 'Combi-Tray'

unit, the security of positive identification can be applied direct to the original sample tube, further ensuring accuracy of sample handling.

*Further information from Francis Hooley, Technicon, Evans House, Hamilton Close, Basingstoke, Hampshire RG21 2YE, UK. Tel.: 0256 29181.*

### H-P Analytical Symposium 1986

The underlying theme of Hewlett-Packard's meeting in Harrogate, UK in June this year was: 'the laboratory of the future'. Throughout the five daily sessions emphasis was placed on the most significant overall development in analytical chemistry today – the growth of robotic sample handling, laboratory automation and information management systems leading to the totally automated laboratory.

Each day of the Symposium covered a specific broad applications area, and examined the instrumental techniques that are used in that field: these were petro-chemistry/energy; general chemicals and environmental; pharmaceutical chemistry; biochemistry/bioscience; and clinical toxicology/forensic science.

Some of the abstracts of papers read during the symposium are reproduced here. There are no plans for publication and further information should be sought from the speakers.

*New techniques and approaches to environmental analysis: Professor J. Miller, Department of Chemistry, Loughborough University of Technology, UK*

The determination of the organic components of environmental samples requires robust, sensitive and selective analytical techniques. In recent years several new methods have been applied with success, and the techniques have become more readily available for everyday use. Immunoassay methods provide convenient and sensitive approaches, and are increasingly used in water analyses and related fields. The advent of fibre optics allows remote sensing and fibre optic systems can probably successfully be combined with immunoassays. Other sensing devices include electrochemical sensors. With some advantages over optical devices. Fluorescence methods are also useful for multicomponent environmental assays, being sensitive and offering several new possibilities. Environmental systems can benefit from the application of modern chemometric methods, such as principal component analysis and pattern recognition, which allow a variety of different multicomponent analyses.

*Flow injection as a sample handling technique for diode array spectrophotometry: Dr Paul Worsfold, Department of Chemistry, The University, Hull, UK*

One of the major attractions of a diode array spectrophotometer, as compared with a conventional scanning UV/visible spectrophotometer, is its rapid data acquisition capability. The usefulness of this feature is considerably diminished if sample preparation and sample introduction are the rate determining steps in the analytical procedure, as is often the case. This presentation described the potential for using flow injection analysis (FIA) as a means of sample introduction and on-line sample treatment for a diode array spectrophotometer (DAS).

An automated FIA-DAS combination was described and potential areas of application discussed. Current work is directed towards the simultaneous determination of three cardiac enzymes in serum samples.

*A new column for the mass spectroscopist: Ultra High Resolution – selectivity tuning of silica capillary columns: Dr Paul Tharagonnet, Marketing Engineer, Columns and Supplies Europe, Hewlett-Packard GmbH, Waldbronn, FR Germany*

Fused silica columns offer the gas chromatographer considerable advantages over conventional packed columns. Generally speaking the greatest advantages of speed of analysis, resolution, sensitivity are achieved by going to very small internal diameters. However, because of limitations imposed by the chromatograph, there are rarely any advantages observed by going to an internal diameter of less than 200μ. There are two application areas where this is not always true.

(1) When using GC/MS when the highest sensitivity and performance of the detector are significantly improved by using the smallest flow rate feasible.
(2) When working with routine samples which contain 50 or less peaks and the desire is for high sample throughput.

In the presentation data were given which showed how the newly introduced 100μ column from Hewlett-Packard performs in these application areas. Data were also presented on the performance characteristics of other new accessories and consumables for the gas chromatographer.

*GC-MS and GC-FT/IR; future partners in the GC laboratory: Dr David Dixon, MS Product Manager, Hewlett-Packard S.A., Geneva, Switzerland*

Of the 'hyphenated' techniques applicable to gas chromatography, both GC-MS and IR-MS have proved to be appropriate with commercial systems available on the marketplace. Of these, GC-MS is presently rather more widespread.

GC-MS, especially in the standard Electron Impact (DI) mode, has provided a wide applications literature and commercial data base collections of 40–80 000 mass spectra. The result has been the ready availability of relatively low-cost quadrupole systems which are to be found in routine use in the GC laboratories. Many of these systems are used for the analysis of 'targetted' compounds, rather than the *a priori* elucidation of structure by spectral interpretation. In the Selected Ion Monitoring Mode (SIM) such systems are also capable of the low pg quantitation which is found of particular value in environmental or pharmokinetic analysis.

Infra-red spectrophotometry is a technique with which most chemists have had rather more experience during their studies. IR instruments are to be found in most teaching laboratories, which is in contrast to MS where samples are often submitted to a central laboratory.

During recent years, the Fourier Transform IR spectrophotometer has proved itself to provide the necessary performance attributes for use with GC, albeit at prices somewhat higher than routine Quadrupole GC-MS. The computation requirement, and particularly those of storage are more demanding than GC-MS but equipment now becomes available at more attractive prices.

MS and IR provide valuable data which is of a complementary nature. IR can readily indicate functional groups and is a method of choice for isomeric characterization. On the other hand, MS provides molecular structure information and molecular weight following interpretation of the mass spectrum. Finally, the information on compound homologs in complex mixtures is readily obtained from GC-MS results.

GC-MS, as mentioned above, enjoys the availability of large libraries of EI spectra. GC-FT/IR in this respect offers limited databases of gas phase spectra generally obtained at a standard eight wavenumber resolution. However, the IR experience under condensed or solid phase conditions is of considerable value. The expected increase in the popularity of GC-FT/IR will certainly result in extension of data bases in the next few years.

GC-FT/IR systems have often shown useable sensitivities 10 to 100x inferior to the GC-MS systems which has certainly reduced effective application to a wide range of analytical problems but this issue has been addressed in the modern systems. GC-FT/IR units are now becoming available which offer sensitivities perhaps only 3–5x away from GC-MS.

GC-IR and GC-FT/IR results were compared and contrasted for modern hardware suitable for use in the GC laboratories. Since the respective data are complementary, some initial approaches for the synergistic combination of results within a GC-FT/IR Data System were discussed.

*Robotics controlled table dissolution testing – successful laboratory automation: Paul Last, Analytical Chemistry Department, Central Research, Pfizer Ltd, Sandwich, Kent, UK*

The past few years have seen an increasing interest in the automation of complex laboratory procedures, but there are still few examples of successful automation using a laboratory robot. Most of these are dedicated to one procedure or series of unit operations.

The dissolution test, which aims to monitor the *in vitro* drug release rate of pharmaceutical dosage forms, is a fundamental test applied during development, stability testing and final QC testing of capsules and tablets. It is a repetitive, labour intensive and time-consuming test which frequently causes 'bottlenecks' in work planning and scheduling. It is additionally a boring and occasionally demotivating test. It is not surprising that this test is one of the first to be evaluated for automation.

The introduction of semi-automated methods, for example flow-through systems, has eased the burden of dissolution testing but only recently has fully automated dissolution testing become available as advances in laboratory robotics have become a commercial reality. A robotic dissolution system has been developed which is routinely used for the dissolution testing of tablets using UV spectrophotometry. The flexibility of the robotic system now allows the testing of other solid dosage forms (for example capsules).

Problems encountered during the validation processes were discussed. The precision and accuracy of the results for the automated test were compared to those of the manual test.

*Biosensors: principles and potential: Dr A. P. F. Turner, Head of Bio-Electronics Division, Cranfield Biotechnology Centre, Cranfield Institute of Technology, Bedford, UK*

A biosensor is a device incorporating a biological sensing element either intimately connected to or integrated within a transducer. The usual aim is to produce a digital electronic signal which is proportional to the concentration of a specific chemical or set of chemicals. The marriage of biology and electronics combines the specificity and sensitivity of systems such as tissues, enzymes, antibodies and DNA with electrochemical, optical calorimetric and acoustic transducers, thus accessing the computing power of the microprocessor. This emerging technology crosses many traditional academic delineations and offers a powerful new tool which threatens to radically alter our attitude to analytical science.

Much of the impetus for work on biosensors has come from medical requirements, particularly in the field of diabetes. In recent years, however, there has been a growing appreciation of the other possible uses of biosensors. Clinical research is spinning off into related veterinary areas and animal husbandry. The food industry is increasingly concerned about quality and has long recognized the value of rapid methods for estimating shelf life, deterioration and contamination. The rise of biotechnology has stimulated investigations into fermentation monitoring and control with possibilities for process control also arising. Concern for both the industrial and natural environment has led to research on sensors for pollutants such as carbon monoxide and herbicides; while the ubiquitous military interest is focusing on specialized needs including biological and chemical defence.

*Details of next year's symposium, which will also be held in Harrogate, from Roger H. Bilder, 65 September Way, Stanmore, Middlesex HA7 2SE, UK. Tel.: 01 954 2105.*

### Chromatography computer extension

Users of the SP4270 or SP4290 Computing Integrators can benefit from a new low-cost, plug-in enhancement which provides the storage and re-integration capabilities of a true multichannel data system. The Memory Module permits integrators to store over 100 average-size LC or GC chromatograms in 256K of RAM without the need for a separate computer. The user may reintegrate any of the stored chromatograms up to 10 times faster than the original integration.

In addition, a highly flexible batch reprocessing mode allows the user to reintegrate and replot any number of stored chromatograms automatically. A 'batch' can be any series of stored chromatograms, and up to 16 different integration parameters, report files, or calibration files.

The Memory Module costs about £850 and is available from stock immediately.

*For more information contact Spectra-Physics, Autolab Division, 17 Brick Knoll Park, St Albans, Herts AL1 5UF, UK. Tel.: 0727 30131.*

### MasterLab

Perkin-Elmer's MasterLab system has expanded its capabilities to meet the needs of the biological laboratory performing repetitive, routine UV/Visible colorimetric bioassays. The modularity of the hardware and software allows a wide variety of bioassays to be performed including protein assays, immunoassays, microtitrations, RIA's and drug binding assays.

The laboratory robot is under the control of the widely used IBM PC and the Perkin-Elmer Robot Language (PERL). A Lambda 3B Spectrophotometer equipped with a Super Sipper, two Master Syringe Units, a Device Interface, and a Master Mixer complete the system components. Spectroscopic measurements, precise liquid handling operations and mixing are all orchestrated by programs written, compiled and executed by PERL.

Robotics allows for automation of manually performed routine laboratory procedures such as the Lowry Assay. Human factors such as imprecise pipetting, operator-to-operator variability and timing variation are virtually eliminated and exposure to hazardous material is substantially reduced.

Use of a commercial software package, such as Lotus 1–2–3, allows data to be analysed by least squares technique. Final protein concentrations are then calculated and data can be plotted in a user-programmable format. Integration with the Perkin-Elmer Laboratory Information Management System (LIMS) offers such additional benefits as extended report generation, quality-control result monitoring and electronic exchange of data among multiple laboratory sites.

Laboratory productivity and efficiency are significantly improved by the versatile MasterLab System interfaced with analytical instruments such as the Lambda 3B Spectrophotometer.

*Further information from Perkin-Elmer Ltd, Post Office Lane, Beaconsfield, Buckinghamshire HP9 1QA, UK.*

### Bench-top sequential ICP system

The introduction by Philips Analytical of a bench-top inductively coupled plasma spectrometer, the PU 7450, means that the company is now able to offer total coverage in atomic spectroscopy. The spectrometer results from a decision to offer a complete choice of instruments. It is a joint venture between Philips Analytical and the manufacturer, Leeman Labs Inc. of Massachusetts, a company chosen for its analytical knowledge, experience and prestige in the market place.

The spectrometer, which bears the Philips label and has been produced to the company's specifications, provides impressive performance at a reasonable price. It permits introduction of ICP into a wide range of industries where the previously high cost of sophisticated ICP systems had been a limiting factor.

The flexibility of the PU 7450 makes it a versatile tool for analysing a wide range of materials, including metals and refractories, geological, mining, environmental, biological and petroleum products.

For routine analysis the spectrometer can determine up to 20 elements in each stored program with a total capacity for three stored programs.

Additional programs may be stored on an external computer; all analytical parameters, including calibration data, are stored in battery-protected memory so that programs can be recalled by a single keystroke.

At the heart of the PU 7450 is an echelle grating monochromator which provides the very high resolution required to separate the complex, line-rich spectra created by the high temperature ICP source.

Most importantly, this high dispersion is achieved without losing efficiency (a problem experienced with most conventional grating spectrometers using extremely narrow slits) because all wavelengths are measured at or very close to the blaze angle.

Measurement accuracy is guaranteed by innovations within the spectrometer, such as the use of fixed optics for simplicity and reliability combined with a moving detector system for unambiguous wavelength selection.

The plasma source is a 27.12 MHz, 2.5 kW tuned oscillator design chosen because of its greater tolerance of variations in sample type. Most organics, for example, are run as easily as aqueous samples. A large torch housing gives good access to the sample introduction assembly.

Various nebulizer/torch options enable the operator to select the best combination for the samples to be analysed.

*Details from Pye Unicam Ltd, York Street, Cambridge CB1 2PX, UK. Tel.: 0223 358866.*

### Chromatography

Two Beckman CALS Systems – the Phase 1 and Phase 2 are now available as a modular approach to computerizing chromatography instruments. The systems feature two recently introduced Beckman products – the MK5 Digimetry Instrument Interface Coupler and PeakPro Chromatography Software.

Phase 1 is scaled for the smaller laboratory, supporting three to 15 instruments and one to four simultaneous users, but includes many of the features of the large-scale products. Full colour graphics, for example, can be displayed and plotted, help is available on-line for inexperienced operators, prompts are user-configurable with user specified defaults, performance can be documented for GLP/GMP compliance and the unique autoranging MK5 interface can be provided for accuracy in signal processing.

PeakPro Software has been designed with input from many chromatographers and will process chromatograms with up to 1000 peaks accurately and efficiently. The new advanced algorithms identify peaks and assign base-lines automatically. Because chromatography is a visual process, PeakPro makes extensive use of graphics to display, examine and construct new methods and analyse data. All this may be done in the interactive mode utilizing a mouse-driven cursor.

Phase 1 can be upgraded to Phase 2 (20 to 30 instruments, eight to 10 simultaneous users) and even further to include 60 linked instruments and/or well proven LIMS software. Original investment is protected at each step. Non-chromatographic instruments can also be supported in these systems.

*Details from Beckman Ltd, Progress Road, Sands Industrial Estate, High Wycombe, Buckinghamshire, UK. Tel.: 0494 41181.*

### Computing integrator

LDC/Milton Roy's CI3000 offers independent processing of two channels of integration with presentation on the graphic display and/or printer/plotter(s). Data are stored in the 512K of internal RAM – a further 32K of memory is assigned for storage of method files (operating parameters) and crucial data which are permanently protected by on-board battery back-up.

The CI3000's interactive graphics software allows data from both channels to be shown simultaneously on the monitor. For post-run processing, a wide range of routines is provided for on-screen editing and manipulation of data. These routines include both scrolling and magnification. The graphics software and a selection of useful application programs are supplied on a plug-in memory module (EPROM).

*Details from LDC (UK) Ltd, Milton Roy House, Diamond Way, Stone Business Park, Stone, Staffordshire ST15 0HH, UK. Tel.: 0785 813542.*

### Technicon

*Magnesium method developed for Technicon Chem 1 Analyser*

Using cyanide-free reagents stable for one year, Technicon researchers have reported the development of a magnesium method for the CHEM 1 capsule chemistry system. Linear to 3.5 mmol/l with within-run CVs of 1·5% and 2·7% at 2·2 and 1·25 mmol/l respectively, the method is based on formation of the red Calmagite-Mg complex at alkaline pH. No significant interference has been observed in samples due to lipaemia, icterus or high urea levels. Negligible interference occurs with calcium at 4·0 mmol/l and heavy metals, correlation studies with atomic absorption show a slope of 0·96, intercept 0·2 and $r = 0{\cdot}992$.

*Improved formulation of urea kits from Technicon*

Urea reagents from Technicon are now prepared using 'dry-blend' techniques which increase stability and ease of preparation. Reagents are stable for one month at 4°C after reconstitution with distilled water.

A choice of three pack sizes is available to ensure minimal wastage for all laboratories – 6 × 20 ml, 6 × 50 ml and 6 × 100 ml, and the method is linear to 43 mmol/l. Adaptation protocols are available for centrifugal analysers, as well as the Technicon RA systems.

*Details from Francis Hooley, Technicon Instruments Company Ltd, Evans House, Hamilton Close, Houndmills, Basingstoke RG21 2YE, UK. Tel.: 0256 52916.*

### China hospitals

Robert S. First's *China Hospital Directory* contains:

Names and address of more than 5000 hospitals in China by bed size.
The speciality of each hospital.
The breakdown of medical doctors in these hospitals by their clinical discipline.
The total hospital admissions and average length of stay, arranged by province.
Number of out-patient visits per hospital.
The total number of hospital beds in China, by province and clinical discipline.

Other demographics such as the population by age group and sex; the healthcare expenditures as a percentage of gross national product; and the major medical problems in China are included in this new directory.

*The cost of the directory is $495. Order by telephoning or writing: Robert S. First, Inc., 707 Westchester Avenue, White Plains, New York 10604, USA; Tel.: 914 949 4248.*

